# Supplementary material for: MetaQTL: a package of new computational methods for the meta-analysis of QTL mapping experiments
Source: BMC Bioinformatics. 2007 Feb 8;8:49. doi: 10.1186/1471-2105-8-49 (PMC1808479; doi:10.1186/1471-2105-8-49)
Supplement: Additional File 7 — MetaQTL Package : jar file and tutorial. This Zip archive contains both the MetaQTL JAR file and the files of the tutorial. [file 1471-2105-8-49-S7.zip › org.inra.metaqtl/doc/org/metaqtl/MetaQtlData.html]

MetaQtlData


|  |  |  |
| --- | --- | --- |
| **Overview** \| **Package** \| **Class** \| **Use** \| **Tree** \| **Deprecated** \| **Index** \| **Help** | |  |
| **PREV CLASS**   **NEXT CLASS** | **FRAMES**    **NO FRAMES**     **All Classes** |  |
| SUMMARY: NESTED \| FIELD \| CONSTR \| METHOD | DETAIL: FIELD \| CONSTR \| METHOD |  |


---


## org.metaqtl Class MetaQtlData

```
java.lang.Object
  org.metaqtl.MetaQtlData
```

---

``` public class MetaQtlData extends java.lang.Object ```

QTL meta analysis first requires to gather the QTL according
to the group trait they belong and then to format the QTL data
into data points to perfom clustering. This class is dedicated
to this task.
> ```
>     Chromosome chrom = ...
>     // Compute the standard deviations of the QTL positions 
>     // on the chromosome.
>     chrom.computeQtlSD(IMetaQtlConstants.SD_MODE_AVAILABLE);
>  
>     MetaQtlData qtlData = new MetaQtlData();
>     qtlData.qtls = chroms.qtls;
>  
>     // First we clusterize
>     // QTL w.r.t to the trait group information
>     qtlData.doTraitGroupClustering();
>     // Then we loop on each trait group 
>     for(int i=0;i<qtlData.ntg;i++) {
>        Double[][] XSD = qtlData.getDataPoints(i);
>        Double[] X     = X[0]; // the qtl positions
>        Double[] SD    = X[1]; // the qtl standard deviations.
>        // do something...
>     }
> ```

---

| **Field Summary** | |
| --- | --- |
| `protected  int` | `ntg`             The number of trait group. |
| `protected  int[]` | `ntgQtls`             The number of QTL in each trait group. |
| `protected  Qtl[]` | `qtls`             The QTL. |
| `protected  int[][]` | `tgidx`             The indices of the QTL in the trait groups. |
| `protected  java.lang.String[]` | `tgnames`             The trait group names. |


**Constructor Summary**

`MetaQtlData(Qtl[] qtls)`
  
          Create a new instance of `MetaQtlData` which
will represent the given array of `Qtl`.


| **Method Summary** | |
| --- | --- |
| `void` | `doTraitGroupClustering()`             According to the values of the QTLTrait.group attribute of the trait attribute of the qtls this methos clusterize the qtls in trait groups. |
| `void` | `doTraitOntologyClustering(IBioOntology ontology)`             According to the given trait ontology this methods clusterize the QTL in group in such a way that each group represents a trait term in the ontology. |
| `java.lang.Double[][]` | `getDataPoints(int tg, boolean ignore)`             For a given trait group indice this methods returns the data points as a matrix of Double X[0..1][0..n-1] where X[0] is the vector of the n position values and X[1] is the vector of the n standard deviations of the positions. |
| `Qtl` | `getQtl(int i, int j)`             Returns the j^th QTL in the i^th cluster. |
| `int[]` | `getQtlIdx(int i)`             Returns the indices of the Qtl for the given trait cluster indice `i`. |
| `int` | `getQtlNumber()` |
| `java.lang.String` | `getTraitClusterName(int i)` |
| `java.lang.String[]` | `getTraitClusterNames()`             Returns the names of the trait clusters. |
| `int` | `getTraitClusterNumber()`             Returns the number of trait groups for this data set as defined by the clustering method. |
| `int` | `getTraitClusterSize(int i)`             Returns the size of the trait cluster i. |
| `void` | `manageMissingData(int opt)`             Manage the missing data. |

**Methods inherited from class java.lang.Object**

`clone, equals, finalize, getClass, hashCode, notify, notifyAll, toString, wait, wait, wait`

**Field Detail**

### qtls

```
protected Qtl[] qtls
```

:   The QTL.

---


### ntg

```
protected int ntg
```

:   The number of trait group.

---


### ntgQtls

```
protected int[] ntgQtls
```

:   The number of QTL in each
    trait group.

---


### tgnames

```
protected java.lang.String[] tgnames
```

:   The trait group names.

---


### tgidx

```
protected int[][] tgidx
```

:   The indices of the QTL in the trait
    groups.


**Constructor Detail**

### MetaQtlData

```
public MetaQtlData(Qtl[] qtls)
```

:   Create a new instance of `MetaQtlData` which
    will represent the given array of `Qtl`.

    **Parameters:**: `qtls` -


**Method Detail**

### doTraitGroupClustering

```
public void doTraitGroupClustering()
```

:   According to the values of the QTLTrait.group
    attribute of the trait attribute of the qtls
    this methos clusterize the qtls in trait
    groups.

---


### getDataPoints

```
public java.lang.Double[][] getDataPoints(int tg,
                                          boolean ignore)
```

:   For a given trait group indice this methods returns
    the data points as a matrix of Double X[0..1][0..n-1]
    where X[0] is the vector of the n position values
    and X[1] is the vector of the n standard deviations
    of the positions.

    :   **Parameters:**: `tg` - the trait group indice **Returns:**: the data points.

---


### doTraitOntologyClustering

```
public void doTraitOntologyClustering(IBioOntology ontology)
```

:   According to the given trait ontology this methods
    clusterize the QTL in group in such a way that each
    group represents a trait term in the ontology.

    :   **Parameters:**: `ontology` - the trait ontology.

---


### getQtlNumber

```
public int getQtlNumber()
```

:   **Returns:**

---


### manageMissingData

```
public void manageMissingData(int opt)
```

:   Manage the missing data.
    `opt` to parametrize the behaviour of the method to
    do the imputation.

    :   **Parameters:**: `opt` - the option for missing data management.

---


### getTraitClusterNumber

```
public int getTraitClusterNumber()
```

:   Returns the number of trait groups for this data set as
    defined by the clustering method.

    :   **Returns:**: the number of trait groups for this data set **See Also:**: `doTraitGroupClustering()`, `doTraitOntologyClustering(IBioOntology)`

---


### getTraitClusterName

```
public java.lang.String getTraitClusterName(int i)
```

---


### getQtlIdx

```
public int[] getQtlIdx(int i)
```

:   Returns the indices of the Qtl for the given
    trait cluster indice `i`.

    :   **Parameters:**: `i` - the indice of the trait cluster. **Returns:**: the indices of the Qtl which belongs to this cluster.

---


### getTraitClusterNames

```
public java.lang.String[] getTraitClusterNames()
```

:   Returns the names of the trait clusters.

    :   **Returns:**: the names of the trait clusters.

---


### getTraitClusterSize

```
public int getTraitClusterSize(int i)
```

:   Returns the size of the trait cluster i.

    :   **Parameters:**: `i` - **Returns:**

---


### getQtl

```
public Qtl getQtl(int i,
                  int j)
```

:   Returns the j^th QTL in the i^th cluster.

    :   **Parameters:**: `i` -: `j` - **Returns:**


---


|  |  |  |
| --- | --- | --- |
| **Overview** \| **Package** \| **Class** \| **Use** \| **Tree** \| **Deprecated** \| **Index** \| **Help** | |  |
| **PREV CLASS**   **NEXT CLASS** | **FRAMES**    **NO FRAMES**     **All Classes** |  |
| SUMMARY: NESTED \| FIELD \| CONSTR \| METHOD | DETAIL: FIELD \| CONSTR \| METHOD |  |


---
